# Characterization of the complete chloroplast genome of leek *Allium porrum* L. (Amaryllidaceae)

**DOI:** 10.1080/23802359.2019.1640090

**Published:** 2019-07-22

**Authors:** Mikhail A. Filyushin, Alexey V. Beletsky, Elena Z. Kochieva

**Affiliations:** Institute of Bioengineering, Research Center of Biotechnology of the Russian Academy of Sciences, Moscow, Russia

**Keywords:** *Allium porrum*, leek, chloroplast genome

## Abstract

The complete chloroplast genome sequence of *Allium porrum* was determined by Illumina single-end sequencing. The complete plastid genome was 152,732 bp in length containing a large single copy (LSC) of 81,744 bp and a small single copy (SSC) of 17,910 bp, which were separated by a pair of 26,524 bp inverted repeats (IRs). A total of 133 genes were annotated, including 80 protein-coding genes, 38 tRNA genes, 8 rRNA genes, and 7 pseudogenes. The overall GC contents of the plastid genome were 36.7%. Unlike *A. sativum* and *A. obliquum* in the leek, plastome *infA* gene is absent, and *rpl23* gene is a pseudogene due to a 4 bp deletion and the formation of a premature stop codon.

Leek (*Allium porrum* L., Amaryllidaceae) due to its taste and valuable dietary properties is a popular vegetable crop, the annual production of which is more than 2 million tons per year (http://www.fao.org/faostat/). In leek, the entire above-ground part is edible – the bleached false stem and green leaves, which are used in food both raw and cooked. At the same time, leek, like other *Allium* species, is a rich source of secondary metabolites (Soininen et al. 2014).

The chloroplast genome *A. porrum* (cultivar ‘Premier’; seed from Federal Scientific Center of Vegetable Crops, Russia) was amplified via long range PCR using 11 pairs of primers developed on the basis of the *Allium cepa* plastid genome (Filyushin, Beletsky, et al. 2018), sequencing was conducted using the Illumina HiSeq 1500 Sequencing System with single-end 220 bp reads. Spades v.3.8 was used to assemble the high-quality short reads into contigs (Bankevich et al. 2012). Contigs were assembled against the complete chloroplast genome *A. cepa* (NC_024813) and *A. sativum* (NC_031829) as a reference. Gaps were closed using assembly graph in Bandage (Wick et al. 2015), reads were then mapped against the resulting single contig to ensure the correctness of the finished assembly. The plastid genome of *A. porrum* was annotated by using the DOGMA program (http://dogma.ccbb.utexas.edu). The start and stop codons for the genes were identified and corrected manually. All pseudogenes were additionally verified by Sanger sequencing from flanking primers.

The assembled *A. porrum* plastid genome (Genbank accession no. MK820026) was 152,732 bp in length, showing a typical quadripartite structure including a pair of inverted repeats (IRs) of 26,524 bp separating one large single copy region (LSC) of 81,744 bp and one small single copy region (SSC) of 17,910 bp. GC contents of the genome were 36.7%. A total of 133 genes were identified that include 80 protein-coding genes, 38 tRNA genes, 8 rRNA genes, and 7 pseudogenes.

Most of the genes are single copy, whereas 17 genes present in double copies, including 6 protein-coding genes (*rps19*, *rpl2*, *ycf2*, *ndhB*, *rps7*), 8 tRNA genes (*trnR-ACG*, *trnL-CAA*, *trnV-GAC*, *trnH-GUG*, *trnI-CAU*, *trnI-GAU*, *trnA-UGC*, *trnN-GUU*) and all 4 rRNA genes in IRs (*rrn4.5*, *rrn5*, *rrn16* and *rrn23*). Intron sequences are found in 17 genes, 15 of which contain a single intron (*atpF*, *rpoC1*, *ndhA*, *trnK-UUU*, *trnG-GCC*, *trnL-UAA*, *trnV-UAC*; 4 genes in IRs: *rpl2*, *ndhB*, *trnI-GAU*, *trnA-UGC*), while two (*clpP* and *ycf3*) have two introns.

Seven genes became pseudogenes due to internal stop codons (*rps2*, *rps16* and *ycf15,* and *rpl23* in IRs) or because of incomplete duplication in the IRB/SSC junction region (*ycf1*). Unlike the *Allium sativum* (Filyushin et al. 2016) and *Allium obliquum* plastomes (Filyushin, Mazur, et al. 2018), in the leek plastome *infA* gene is absent (additionally verified by Sanger sequencing).

On the ML tree, *A. porrum* clustered with other *Allium* species, the closest to it is garlic *A. sativum*, with which they belong to the section Allium (Figure 1).

**Figure 1.**
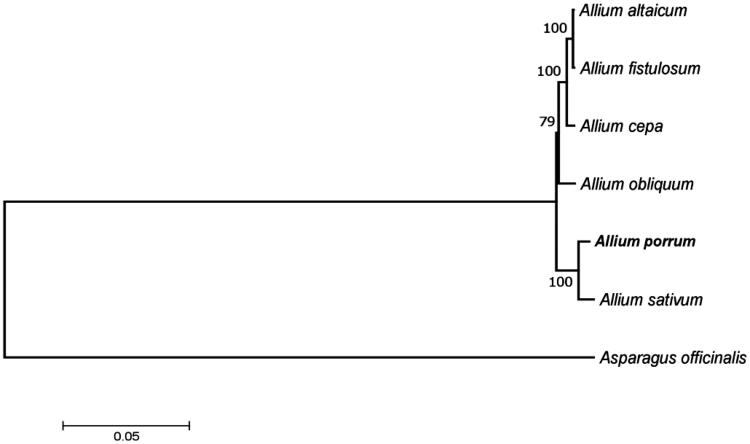
Phylogenetic tree inferred by maximum-likelihood using 80 protein-coding gene sequences from plastomes of *Allium* species (*Allium altaicum* (NC_040972), *Allium cepa* (NC_024813), *Allium fistulosum* (NC_040222), *Allium obliquum* (NC_037199), *Allium porrum* (MK820026), *Allium sativum* (NC_031829)) and *Asparagus officinalis* (NC_034777) as an outgroup. PhyML 3.1 was used for the sequence alignment and construction of the tree. Bootstrap support values based on 1000 replicates are displayed on each node.
